# DMENet: Diabetic Macular Edema diagnosis using Hierarchical Ensemble of CNNs

**DOI:** 10.1371/journal.pone.0220677

**Published:** 2020-02-10

**Authors:** Rajeev Kumar Singh, Rohan Gorantla

**Affiliations:** Department of Computer Science, Shiv Nadar University, Noida, UP, India; Politechnika Krakowska im Tadeusza Kosciuszki, POLAND

## Abstract

Diabetic Macular Edema (DME) is an advanced stage of Diabetic Retinopathy (DR) and can lead to permanent vision loss. Currently, it affects 26.7 million people globally and on account of such a huge number of DME cases and the limited number of ophthalmologists, it is desirable to automate the diagnosis process. Computer-assisted, deep learning based diagnosis could help in early detection, following which precision medication can help to mitigate the vision loss. Method: In order to automate the screening of DME, we propose a novel DMENet Algorithm which is built on the pillars of Convolutional Neural Networks (CNNs). DMENet analyses the preprocessed color fundus images and passes it through a two-stage pipeline. The first stage detects the presence or absence of DME whereas the second stage takes only the positive cases and grades the images based on severity. In both the stages, we use a novel Hierarchical Ensemble of CNNs (HE-CNN). This paper uses two of the popular publicly available datasets IDRiD and MESSIDOR for classification. Preprocessing on the images is performed using morphological opening and gaussian kernel. The dataset is augmented to solve the class imbalance problem for better performance of the proposed model. Results: The proposed methodology achieved an average Accuracy of 96.12%, Sensitivity of 96.32%, Specificity of 95.84%, and F−1 score of 0.9609 on MESSIDOR and IDRiD datasets. Conclusion: These excellent results establish the validity of the proposed methodology for use in DME screening and solidifies the applicability of the HE-CNN classification technique in the domain of biomedical imaging.

## Introduction

Diabetic Macular Edema (DME) is a complication of Diabetic Retinopathy (DR), and it usually occurs when vessels in the central part of the macula are affected by the fluid accretion [1]. DME is caused due to diabetes which is a chronic disease induced by inherited and/or acquired deficiency in the production of insulin by the pancreas. DME is an advanced stage of DR that can lead to irreversible vision loss [2–4].

Diabetes currently affects more than 425 million people worldwide and is expected to affect an estimated 520 million by 2025. It is estimated that 10% of people who suffer from some form of Diabetes are at the risk of DME. DME currently affects around 26.7 million people globally, and the number is expected to rise to around 50 million by 2025 [3, 5]. About 7.7 million Americans have DR and approximately 750,000 are suffering from DME. [4].

Identifying exudates in fundus images is a standard technique for determining DME [6, 7]. The nearness of exudates to the macula determines the severity of DME and the probability of DME is higher when the exudates are closer to the macula with the risk being maximum when they are inside the macula [8]. Immediate treatment is required if a person is diagnosed with DME so as to avoid complete vision loss.

The ratio of ophthalmologists to population in developed countries like USA is 1:15,800 and in developing countries like India is 1:25,000 in urban areas and 1:219,000 in rural areas [9, 10]. The limited number of ophthalmologists cannot keep up with the rapidly increasing number of DME patients and this heavily skewed ratio of ophthalmologists with respect to DME patients is also leading to delayed services to the needy. Manual evaluation of DME is not adaptable in a large-scale screening scheme, especially in developing countries where there is a shortage of ophthalmologists [11]. One out of nine individuals turned out to be positive DME cases during the screening tests as mentioned in [12]. Another challenging aspect of the healthcare sector in the developing countries is to provide the correct and timely treatment at an affordable cost. In this kind of a scenario, we require an automated disease discovery framework that can reduce cost and workload, as well as manoeuvre the shortage of ophthalmologists by limiting the referrals to those cases that require prompt consideration. The reduction of effort and time of ophthalmologists in diagnosis will be pivotal for arresting the growth of DME cases.

Propelled by these promising possibilities, we propose to develop an effective solution using Deep learning techniques to automatically grade the fundus images. Machine learning techniques have powered many aspects of medical investigations and clinical practices. Deep learning is emerging as a leading machine learning tool in computer vision and has started to command significant consideration in the field of medical imaging. Deep learning techniques and in particular, convolutional neural networks, have rapidly gained prominence for analysis of the medical images.

In this paper, we propose a novel algorithm DMENet which automatically analyses the preprocessed color fundus images. Preprocessing on the images is performed using morphological opening and gaussian kernel. The dataset is augmented to solve the class imbalance problem for better performance of the proposed model. After Preprocessing, the images are passed through a two-stage pipeline. In the first stage, the algorithm detects the presence/absence of DME and once the presence of DME is confirmed it is passed through the second stage where the image is graded based on the severity. Both these stages are equipped with the proposed state-of-art technique called Hierarchical Ensemble of Convolutional Neural Networks (HE-CNN).

The proposed DMENet algorithm in this paper is built on a novel classification structure known as HE-CNN which uses the concept of *ensemble learning*. Ensemble learning is a technique which combines the outputs from multiple classifiers to improve the classification performance of the model [13]. This approach is intuitively used in our daily lives, where we seek guidance from multiple experts, weigh and combine their views to make a more informed and optimized decision. In matters of great importance that have financial or medical implications, we often seek a second opinion before making a decision, for example, having several doctors agree on a diagnosis reduces the risk of following the advice of a single doctor whose specific experience may differ significantly from that of others.

The major contributions of the proposed work can be summarized as follows:

- A preprocessing technique to enhance the key features of the raw fundus images by reducing noise in the background.
- A two stage DMENet pipeline which is first of its kind and performs disease classification as well as severity grading.
- A novel ensemble technique HE-CNN which performs image classification effectively by tackling the problem of overfitting, a common problem in the biomedical image classification tasks.
- A new loss function is designed to train the HE-CNN ensemble using L2-loss to boost the classification performance.

The next section of **Related Work** gives an outline of the existing techniques for DME diagnosis. The section on **Materials and Preparation** presents the details of the datasets and the data preparation strategies employed. A detailed description of DMENet is given in **Proposed Methodology** section. **Experiments and Results** section provides the implementation details along with a clear analysis of the results obtained. A succinct overview of the proposed research as well as future scope is given in the **Conclusions** section.

## Related work

The feasibility of manual assessment for the diagnosis of ophthalmic cases has become practically untenable. Automation of at least the first level diagnosis is a clear requirement to improve the efficacy, affordability, and accessibility of our healthcare system. Over the last two decades, many research groups have extensively worked on automating the diagnosis of ophthalmic problems using color fundus and OCT images. Image acquisition, processing, and diagnosis using color fundus images are comparatively faster and in case of developing nations where the cost factor plays a vital role, color fundus imaging scores higher than others. Recently the use of data-driven machine learning and deep learning techniques on classical expert labeled image analysis have gained prominence. Convolutional Neural Networks have become the method of choice for automated grade assessment of DME. The techniques used in previous works on automated DME grading using color fundus images can be broadly categorized as (a)Feature detection and classification using hand-crafted techniques (b)Combination of hand-crafted and machine learning techniques (c)Employing Deep learning techniques especially CNNs for feature extraction and classification.

One of the earliest automated systems using handcrafted technique given by Siddalingaswamy and Gopalakrishna [14] used clustering and mathematical morphological techniques to detect exudates. In [15] the authors adopted marker-controlled watershed transformation for extracting exudates to perform DME stage classification. The work given in [16] used top-down image segmentation and local thresholding to find the region of interest, followed by exudate detection. Deepak and Jayanthi [11] used a supervised learning approach to capture the global characteristics in fundus images and assessed disease severity using a rotational asymmetric metric by examining the symmetry of the macular region. The work proposed in [17] used a combination of handcrafted and machine learning techniques. This work detected macula by locating darkest pixel along the enhanced blood vessels followed by clustering of pixels where the largest cluster was considered as the macula. They used Gabor filter as well as adaptive thresholding to detect exudates and graded the DME severity with the help of a support vector machine (SVM). The work given in [18] proposed a method for texture extraction from various regions of interest of the fundus image and employed SVM for grading of DME.

However, the overall performance of the above-described grading systems is largely based on feature extraction strategies, exudate segmentation and the location of anatomical structures i.e. macula and fovea. In addition, the extraction of features is hugely dependent on the dataset being used in the methodology. Finding and developing a feature set that is suitable for different datasets remains a challenge. Thus deep learning emerged as a more promising approach to learn features automatically. One of the most recent works [19] used CNNs to automatically extract features and grade the input fundus images. The authors obtained an accuracy of 88.8%, sensitivity of 74.7% and specificity of 96.5% on MESSIDOR dataset [19].

One of the pioneering works in the domain of ensemble learning was proposed by Dasarthy and Sheela [20] which deals with the partitioning of feature space using two or more classifiers. Recently ensemble learning techniques applied on computer vision problems in the domain of medical imaging with CNNs as classifiers demonstrated significant improvement in the performance [21, 22]. Feature space expansion and use of different starting points have been instrumental in providing a better approximation of the optimal solution and has been cited as the rationale for the improved classification using ensemble learning. Ensemble learning offers a promising alternative to the currently used techniques in the computer vision domain and we wish to explore it further in the proposed DMENet algorithm.

## Materials and preparation

### Datasets

**Fig 1:**
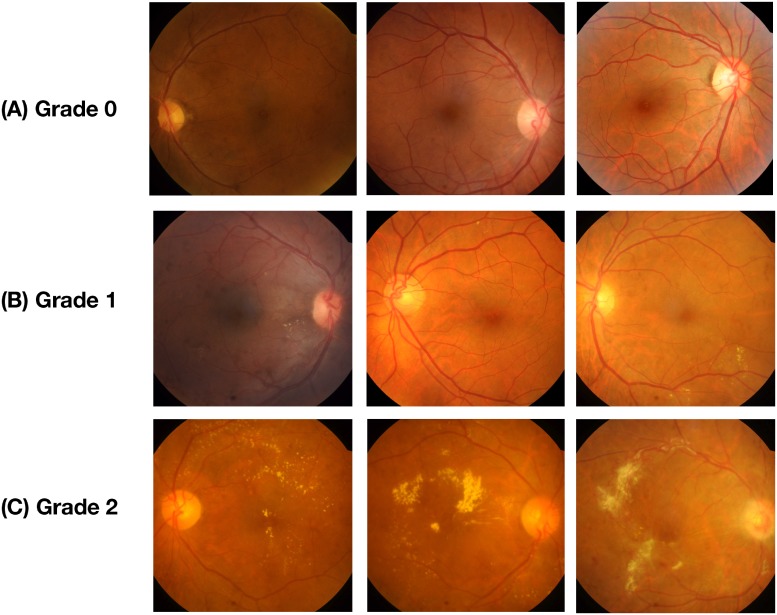
Representative fundus images with different pathological severity of DME. (A) Denotes the images of Grade 0 severity which have no hard exudates, (B) Represents Grade 1 severity which has exudates outside the radius of one disc diameter from the macula center, and (C) Denotes the fundus under the Grade 2 severity category, with exudates within the radius of one disc diameter from the macula center.

The proposed model was trained using data from two publicly available datasets, IDRiD [23] and MESSIDOR [24]. Both these datasets are graded on a scale of three, where each grade is described as given in Table 1. Fig 1 gives the visual depiction of the various severity stages of DME.

**Table 1:** Description of DME severity grading.

Grade	Description
0	No Apparent hard Exudate(s)
1	Presence of hard Exudate(s) outside the radius of one disc diameter from the macula center
2	Presence of hard Exudate(s) within the radius of one disc diameter from the macula center

#### IDRID

This database contains 516 fundus images. These images were captured by a retinal specialist at an Eye Clinic located in Nanded, Maharashtra, India. Experts have verified that all images are of adequate quality, are clinically relevant, no image is duplicated and a reasonable mixture of disease stratification representative of DME is present. The images have a resolution of 4288×2848 pixels. The dataset contains 222 image of grade 0, 41 of grade 1 and 243 images of grade 2. Images were acquired using a Kowa VX-10 alpha digital fundus camera with a 50° field of view (FOV), and all are centered near to the macula.

#### MESSIDOR

The Messidor database has been established to facilitate studies on computer-assisted diagnoses of diabetic retinopathy. The database contains 1200 fundus images acquired at three different locations. The images have resolutions of 1440*960, 2240*1488 and 2304*1536 pixels. This dataset contains 974, 75 and 151 images of grades 0, 1 and 2 respectively. Images were acquired using a color video 3CCD camera on a Topcon TRC NW6 non-mydriatic fundus camera with a 45° FOV.

### Data preparation

#### Data augmentation

**Fig 2:**
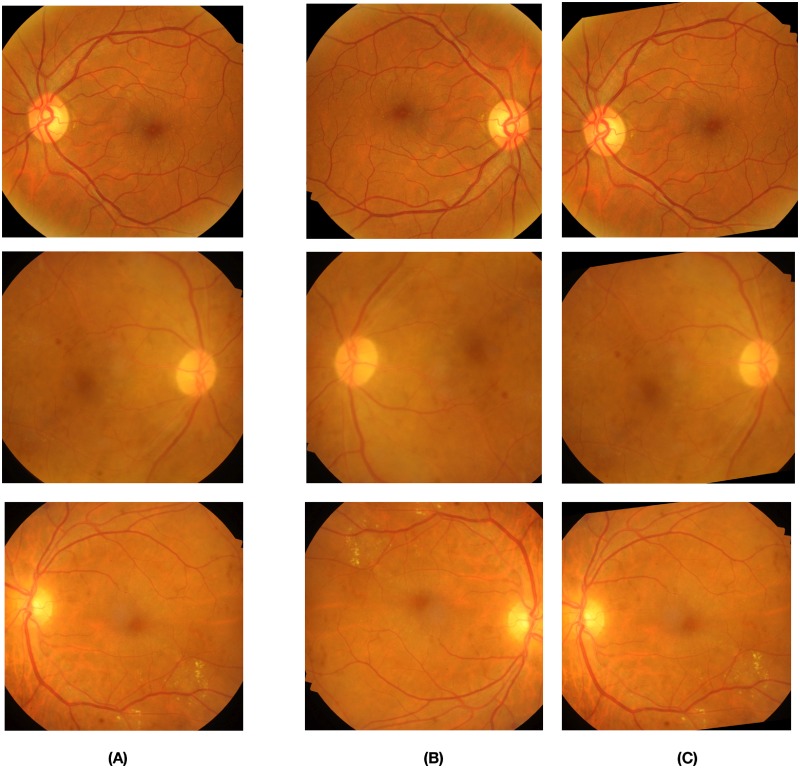
Reprenstative fundus images depicting our augmentation techniques. The images marked under column (A) are the original fundus images that are to be augmented. The fundus images under columns (B), (C) are the images generated owing to flipping and random rotations ranging from 10° to 40° respectively.

In order to build a robust automated disease grading system using CNNs, it is important to have a dataset of images with uniform representation from all the classes. Datasets are often imbalanced in the field of medical imaging, as the number of patients that turn out to be positive is much lower than the total number of cases. This class imbalance problem introduces significant challenges when training deep CNNs which are data-intensive [25]. Another common issue that occurs while training deep CNNs on smaller datasets is overfitting [26]. Training deep CNNs on larger datasets has shown to improve the robustness and generalisability of the model [27]. Data augmentation is an effective solution to reduce overfitting during CNN training as well as to balance the samples across different classes. Various techniques of augmentation, such as Flips, Gaussian-Noise, Jittering, Scaling, Gaussian-Blur, Rotations, Shears, etc., are commonly used. Flips, Rotations, and Scaling have outperformed other techniques [4]. Our augmentation methods include random rotations between 10° to 40° and horizontal flips. The visual examples of our augmentation techniques can be seen in Fig 2.

#### Data preprocessing

Preprocessing plays a key role in improving the accuracy of results by reducing noise in the background. It removes some of the variations between images due to differing lighting conditions and camera-resolution thus making the data consistent [27]. The images contained in the above-mentioned databases have different resolutions which are scaled down to a fixed size to create a standardized dataset. The standardized dataset obtained is preprocessed to improve image contrast which would help in the clear distinction of exudates from the background. The preprocessed image is obtained in a two-step process where we first perform image enhancement and follow it up with the application of the morphological opening operation on the output of the first step. The enhancement of the input image is based on the mathematical formula given in Eq 1.

$$\begin{array}{c} U_{en}\left(x,y;\sigma \right)=\lambda G\left(x,y;\sigma \right)*U\left(x,y\right)+\omega U\left(x,y\right)+\delta \end{array}$$ (1)

where *U*_*en*_ denotes the corresponding output image after enhancement, *U*(*x*, *y*) denotes the raw fundus image, *G*(*x*, *y*; *σ*) is a Gaussian kernel with scale *σ* [27]. The convolution operator is represented by *. In this paper *σ* is empirically set as 25, λ, *ω* and *δ* are parameters to control the weights, which are empirically set as -4, 4 and 0.5. Upon conducting a number of experiments, these values were determined and validated by an ophthalmologist.

**Fig 3:**
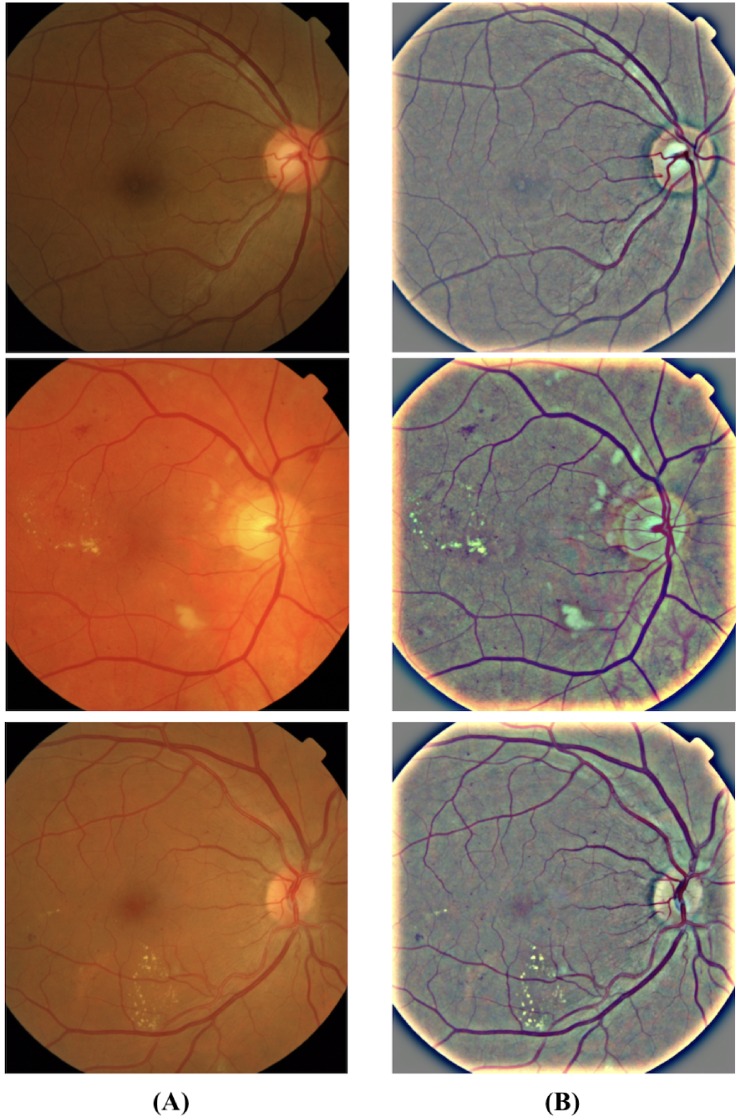
Representative fundus images showing a clear distinction between original and preprocessed images. The images in the column (A) are the raw fundus images and the images in the column (B) show the preprocessed images wherein we can observe the exudates (white/pale yellow spots) and blood vessels more clearly with the controlled background noise.

Morphological opening is performed on the enhanced image to control the brightness of blood vessels which appear brighter than other retinal surfaces due to the lower reflectance [25]. It performs the next step using the expression given by Eq 2.

$$\begin{array}{c} U_{p}=U_{en}\left(x,y;\sigma \right)⚬\varphi \end{array}$$ (2)

where *U*_*p*_ denotes the corresponding output image after performing morphological opening and is the final output of the preprocessing stage. *φ* represents the structuring element disk with a radius of 3 pixels and ⚬ denotes a morphological opening operation. Fig 3 shows the visual representation of our preprocessing technique discussed above.

## Proposed methodology-DMENet

**Fig 4:**
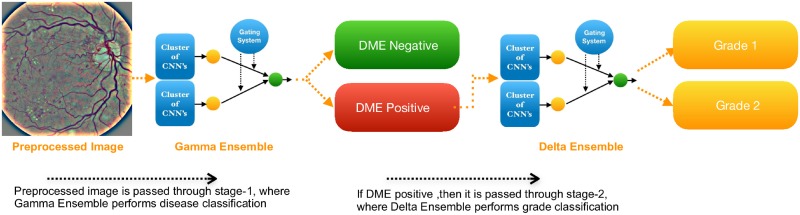
Illustration of the workflow of proposed model—DMENet. DMENet is a two-stage pipeline, where, in the first stage, the algorithm detects the presence/absence of DME and once the presence of DME is confirmed it is passed through the second stage where the image is graded based on the severity.

The description of the proposed methodology- DMENet is presented in this section. DMENet is a two-stage pipeline that performs disease classification (Positive DME and Negative DME) in the first stage followed by severity grading (Grade 1 and Grade 2) in the second stage. Only those images that are classified as positive DME are passed onto the second stage. The workflow of our algorithm is illustrated in Fig 4. Each stage comprises of a classification structure (referred to as Gamma ensemble in the first stage and Delta ensemble in the second stage) based on our proposed novel ensemble technique known as *Hierarchical Ensemble of Convolutional Neural Networks* (HE-CNN). To delve deeper into the ensemble methodology, it is prudent to understand CNNs architecture and fine-tuning techniques as given below.

### Background: CNN and fine-tuning

CNNs have been used in biomedical image analysis since the 1990’s [28, 29], however, with the advent of GPUs and the availability of larger and better datasets, they have started showing superior performance. The strength of CNN lies in its deep architecture which is responsible for extracting distinguishing features at different layers of abstraction [30, 31]. CNNs are fundamentally made of three types of layers, namely convolutional, pooling, and fully-connected layers. The convolutional layer is composed of a set of convolutional kernels that are responsible to learn the patterns or specific features from the input. These kernels compute different feature maps and each neuron in a feature map is associated with a region of neighboring neurons of the previous layer. The new feature map can be obtained by convolving the input with a trained kernel followed by application of element-wise nonlinear activation function on the results obtained using a convolution operator [32]. Activation functions are extremely important for CNNs to learn and comprehend non-linear complex functional mapping between the input and the response variable. The pooling layer is placed between two convolutional layers with the objective of reducing spatial dimensions, improving the computing performance and reducing the number of parameters. There are various types of pooling operations, however, the most commonly used ones are max-pooling [33], and average pooling [34]. After several convolutional and pooling layers, a fully connected layer that performs high-level reasoning is obtained [35, 36]. A fully connected layer takes all the neurons in the previous layer and connects them to each of the current layer’s single neurons to generate global semantic information. [32].

Various studies [37, 38] have established that transfer learning or fine-tuning a CNN is better than training from scratch. Training CNNs from scratch suffers from several issues especially the requirement of having a large dataset is a serious concern in the medical domain where expert annotation is a costly affair. Training a CNN from scratch is also computationally expensive, time-consuming and suffers from frequent overfitting and convergence issues. An effective alternative for training a CNN is transfer learning. Transfer learning is a method where CNNs first learn features in one setting and then adapt and applies it in another setting. The factors which affect the transfer learning strategy is the size of the new dataset on which it is applied and its similarity to the original dataset [39]. CNNs learn low-level features in the early layers which are general for any network whereas high level features are learned in later layers that are dataset dependent. One of the more advanced approaches of transfer learning is Fine-tuning which employs the back-propagation algorithm to update the pre-trained weights *w* of a CNNs. Fine-tuning is an iterative process that works by minimizing the cost function with respect to the pre-trained weights [22]. The cost function is represented as follows

$$\begin{array}{c} C\left(w,D\right)=\frac{1}{n}\sum_{j=1}^{n}l\left(f\left(D_{j},w\right),\hat{c_{j}}\right) \end{array}$$ (3)

where *D* is training dataset with n images, *D*_*j*_ is the *j*^*th*^ image of *D*, *f*(*D*_*j*_, *w*) is the CNN function that predicts the class *c*_*j*_ of *D*_*j*_ given *w*,$\hat{c_{j}}$ is the ground-truth of *j*^*th*^ image, $l(c_{j},\hat{c_{j}})$ is a penalty function based on logistic loss *l* for predicting *c*_*j*_ instead of $\hat{c_{j}}$.

In order to minimize the cost function, the Stochastic Gradient Descent algorithm [40] is commonly used where the cost over entire training dataset is calculated based on the approximated results obtained over mini-batches of data. If $w_{m}^{i}$ denotes the weights in the *m*^*th*^ convolutional layer at iteration *i*, and $\hat{C}$ denotes the cost over a mini-batch of size *y*, then the updated weights in the next iteration are computed as follows [38]

$$\begin{array}{c} w_{m}^{i+1}=w_{m}^{i}+v_{m}^{i+1} \end{array}$$ (4)

$$\begin{array}{c} v_{m}^{i+1}=\eta v_{m}^{i}-\tau^{i}\alpha_{m}\frac{\partial \hat{C}}{\partial w_{m}} \end{array}$$ (5)

$$\begin{array}{c} \tau^{i}=\tau^{\left\lfloor \frac{iy}{\left|Y\right|}\right\rfloor } \end{array}$$ (6)

where |*Y*| denotes the number of training images, *α*_*m*_ is the learning rate of the *m*^*th*^ layer which controls the size of updates to the weights, *η* is the momentum coefficient that indicates the contribution of the previous weight update in the current iteration. This has the effect of speeding up the learning process while simultaneously smoothing the weight updates, *τ* is the scheduling rate that decreases learning rate *α* at the end of each epoch.

### Proposed classification methodology- Hierarchical Ensemble of Convolutional Neural Networks

**Fig 5:**
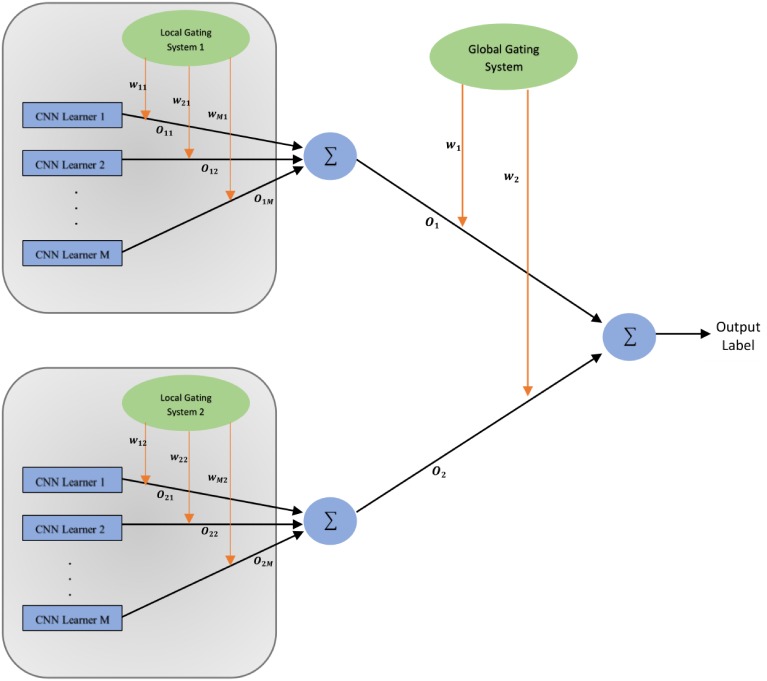
Illustration of the proposed HE-CNN model (Two-level architecture).

This section introduces the proposed novel classification structure known as Hierarchical Ensemble of Convolutional Neural Networks (HE-CNN) which is used to design the Delta and Gamma ensembles present in the DMENet pipeline as shown in Fig 4. An overview of the HE-CNN is shown in Fig 5 (Fig 5 shows HE-CNN representation of a two-level architecture, however, this methodology can be generalized for *n* levels). The proposed HE-CNN architecture comprises multiple learners that maps the input to the output and contains various Gating Systems that characterize the hierarchical structure of the architecture. There is a Local Gating System (LGS) for each cluster of learners and a Global Gating System (GGS) that serves to consolidate the outputs of these clusters. The output of the *i*^*th*^ cluster is given by

$$\begin{array}{c} O_{i}=\sum_{j}w_{ji}O_{ij} \end{array}$$ (7)

where *w*_*ji*_ is activation of *j*^*th*^ output unit of Local Gating System in the *i*^*th*^ cluster and *O*_*ij*_ denotes the output given by *j*^*th*^ learner in the *i*^*th*^ cluster. The final output of the HE-CNN architecture is given by

$$\begin{array}{c} O=\sum_{i}w_{i}O_{i} \end{array}$$ (8)

where *w*_*i*_ is activation of *i*^*th*^ output unit of Global Gating System and *O*_*i*_ denotes the output given by *i*^*th*^ cluster. The outputs of the gating systems are normalised using softmax function [41]

$$\begin{array}{c} w_{ji}=\frac{\text{exp}({\text{g}}_{\text{ji}})}{\sum_{\text{m}}\text{exp}\left({\text{g}}_{\text{mi}}\right)} \end{array}$$ (9)

and

$$\begin{array}{c} w_{i}=\frac{\text{exp}({\text{g}}_{\text{i}})}{\sum_{\text{k}}\text{exp}\left({\text{g}}_{\text{k}}\right)} \end{array}$$ (10)

where *g*_*i*_ and *g*_*ji*_ are the weighted sums at the output units of the corresponding gating networks.

As shown in Fig 5, in this architecture we divide the input space into a nested set of regions and then attempt to fit simple surfaces to the data which fall in these regions. The regions would be having soft boundaries, implying that the data points are spread across multiple regions and the boundaries between these regions are simple parameterized surfaces which are adjusted from the learning process [42]. Every learner has expertise in one specific area of the high-dimensional input space and each learner estimates the conditional posterior probability on the partitioned feature space separated by a gating system based on the given input. This model combines the outputs of several CNN learners by training the gating system. The gating system in the architecture are basically classifiers responsible for dividing the input space. The decision of division is based on the learners capability to model the input-output functions within their respective regions as quantified by their posterior probabilities. The hierarchical nature of this architecture is analysed using probabilistic interpretation as given below. This section also addresses the error functions used to train both CNN learners and gating systems.

#### Probabilistic interpretation

The HE-CNN model learns from each cluster which have multiple CNN learners that are experts in their respective regions. Each *j*^*th*^ learner in *i*^*th*^ cluster maps the input *x* to one of the output classes *G* (In Gamma Phase-DME or No DME and in Delta Phase-Grade 1 or Grade 2). The total probability of generating *G* from x is the mixture of the probabilities of generating *G* from each of the component densities *P*(*G*, *x*; *O*_*ij*_), where the mixture components are multinomial probabilities:

$$\begin{array}{c} P\left(G,x\right)=\sum_{i}w_{i}\sum_{j}w_{ji}P\left(G,x;O_{ij}\right) \end{array}$$ (11)

Here *P*(.) represents the likelihood function of the HE-CNN model. The gating system is responsible for assigning weights and thereby allowing the overall model to execute a competitive learning process by maximizing the likelihood function of the training data [43–45]. Each learner module specializes in exclusive regions of feature space and all modules in this methodology learn simultaneously by interacting with each other rather than learning independently [46]. The learners in a cluster are closely linked with each other and learn similar mappings early in the training phase. They differentiate later in training as the probabilities associated with the cluster to which they belong become larger. Thus the architecture tends to acquire coarse structure before acquiring fine structure. This particular feature of the architecture is notable as it provides robustness to overfitting problem in deep hierarchies.

#### Error function

Taking the error functions used in [42, 44], suitable changes are made to adapt to the proposed model. Assuming a training set of *V* images, the error function of CNN learners for *v*^*th*^ input image *x*_*v*_ is defined as:

$$\begin{array}{c} e_{learner}^{v}=-\text{ln}\;(\sum_{i}w_{i}\sum_{j}w_{ji}\;\text{exp}\;(-\frac{1}{2}{\left\|D^{v}-O_{ji}^{v}\right\|}^{2})) \end{array}$$ (12)

where *D*^*v*^ is the desired output vector, $O_{ji}^{v}$ is the output vector of *j*^*th*^ learner in *i*^*th*^ cluster. The effective error for *j*^*th*^ learner in *i*^*th*^ cluster on full training set is defined as

$$\begin{array}{c} E_{ji}=\sum_{v=1}^{V}h_{ji}^{v}\left(D^{v}-O_{ji}^{v}\right) \end{array}$$ (13)

here $h_{ji}^{v}$ is the posterior probability estimate provided by *j*^*th*^ learner of *i*^*th*^ cluster for input *x*_*v*_. $h_{ji}^{v}$ is given as

$$\begin{array}{c} h_{ji}^{v}=\frac{w_{i}\sum_{j}w_{ji}\;\text{exp}\;(-\frac{1}{2}{\left\|D^{v}-O_{ji}^{v}\right\|}^{2})}{\sum_{i}w_{i}\sum_{j}w_{ji}\;\text{exp}\;(-\frac{1}{2}{\left\|D^{v}-O_{ji}^{v}\right\|}^{2})} \end{array}$$ (14)

The error functions of both the gating systems (GGS, LGS) is defined as follows

$$\begin{array}{c} E_{GGS}=\frac{1}{2}\sum_{v=1}^{V}{\left\|h_{ji}^{v}-w_{i}^{v}\right\|}^{2} \end{array}$$ (15)

$$\begin{array}{c} E_{LGS}=\frac{1}{2}\sum_{v=1}^{V}{\left\|h_{j}^{v}-w_{j}^{v}\right\|}^{2} \end{array}$$ (16)

where $h_{j}^{v}$ gives the posterior probability provided by *j*^*th*^ learner in the cluster for which error function is being calculated. It is defined as given below.

$$\begin{array}{c} h_{j}^{v}=\frac{w_{j}^{v}\;\text{exp}\;(-\frac{1}{2}{\left\|D^{v}-O_{j}^{v}\right\|}^{2})}{\sum_{j}w_{j}^{v}\;\text{exp}\;(-\frac{1}{2}{\left\|D^{v}-O_{j}^{v}\right\|}^{2})} \end{array}$$ (17)

## Experiments, results and discussion

### Evaluation metrics and experimental strategy

To assess the performance of the proposed DMENet methodology and HE-CNN ensemble, standard performance measures have been employed. The Confusion Matrix and Receiver Operating Characteristic (ROC) analyses are used to calculate the accuracy, precision, sensitivity/recall, specificity and average area under the ROC curves (AUC). F1−score is the harmonic mean of precision and recall [47]. F1−score is calculated as given below.

$$\begin{array}{c} F1-score=2*\frac{Precision*Recall}{Precision+Recall} \end{array}$$ (18)

F1−score is a more robust metric to evaluate the classification performance as it takes into consideration the class imbalance problem by giving equal importance to precision and recall, thus involving both false positives and false negatives. F1−score ranges between 0 and 1, reaches the best value of 1 when the balance between precision and recall is perfect. Cohen’s kappa (*κ*) score is used to determine the potential of HE-CNN in partitioning the feature space [48]. *κ* measure signifies the level of agreement between classifiers and kappa value ranges between −1 and 1 (higher the *κ* score, better is the agreement).

In this study, a 5-fold cross-validation technique is applied to assess the classification measures and to generalize the performance of the model. The dataset is roughly split into five equal-size partitions while ensuring that each partition has good representation as a whole. By preserving random seed across all iterations, the dataset-dependent bias is eliminated. Four partitions are used for training and the rest of the partition were used for testing. This step has been repeated five times until all the different test set choices have been evaluated. Over the five folds, the classification measures are averaged.

### Experiment design

This study is based on a two-stage DMENet methodology which uses HE-CNN as a key classification algorithm in each stage. One of the main issues while developing the DME screening solution is the class-imbalance problem. The datasets being used contains a significant portion of DME negative images and more importantly the grade 1 images are less than 10% of the whole dataset. This can lead to mis-classification of an image with grade 1 characteristics. To circumvent this issue the tri-class (Grade 0, 1, 2) classification problem is converted into a binary classification problem using two stage DMENet model as depicted in Fig 4. We have used a combination of pre-trained networks like ResNet [49], DenseNet [50], SqueezeNet [51], GoogleNet [31] and SE-ResNet [52] as the learners in each cluster of the ensemble model. The gating systems, both the local and global are based on the CNN architectures given in Table 2. The final implementation details of each classification structure (i.e. Gamma and Delta Ensemble) after performing number of experiments are given below.

**Table 2:** CNN structures.

Structure	Input Size	Number of layers	First Convolution mask	Other Convolutional masks	Max-Pooling Size	Stride	Pool type in FC	Number of output neurons
CNN-1	224 x 224	18	5 x 5	3 x 3	2 x 2	2	Average	2
CNN-2	224 x 224	22	5 x 5	3 x 3	2 x 2	2	Average	2
CNN-3	224 x 224	28	7 x 7	3 x 3	2 x 2	2	Average	2
CNN-4	224 x 224	32	7 x 7	3 x 3	2 x 2	2	Average	2
CNN-5	224 x 224	40	7 x 7	3 x 3	2 x 2	2	Average	2

*Gamma Ensemble* follows the *two-level* HE-CNN architecture as shown in Fig 5. The CNN learners in the first cluster are setup using a combination of ResNet and DenseNet architectures. pre-trained ResNet-50 architecture is used as learner-1 and pre-trained DenseNet-161 architecture as learner-2. The outputs of these learners were aggregated using LGS whose architecture is based on the CNN-1 structure as given in Table 2. The second cluster is composed of three CNN learners based on SqueezeNet, SE-ResNet and ResNet architectures. A pre-trained SE-ResNet-50 architecture is used as learner-1, pre-trained SqueezeNet as learner-2 and pre-trained ResNet-34 architecture as learner-3. The CNN-2 structure given in Table 2 is used as LGS for aggregating the outputs. In order to combine the outputs of both these clusters, we used a GGS which used CNN-3 architecture as given in Table 2.

*Delta Ensemble* is based on *three-level* HE-CNN architecture. It contains an additional cluster of CNN learners and corresponding LGS as compared to the two-level architecture of Gamma Ensemble. The first cluster contains two pre-trained CNN learners which are based on the architectures of DenseNet and SE-ResNet. A DenseNet-169 architecture is used as learner-1, SE-ResNet-50 as learner 2. The outputs of these learners are combined using an LGS-1 which used CNN-2 architecture as given in Table 2. The two pre-trained CNN learners in the second cluster are based on architectures of ResNet-50 and SqueezeNet. CNN-1 architecture given in Table 2 is employed as LGS-2 to combine the outputs of the learners in the second cluster. The third cluster is composed of three pre-trained CNN learners- DenseNet-161, ResNet-34 and GoogLeNet. The CNN-3 architecture is employed as LGS-3 and the CNN-4 architecture is employed as GGS demonstrated in Table 2.

All the CNN learners were initialized on the ImageNet [53] dataset weights. The filter weights derived from ImageNet were then finetuned through back-propagation thus minimizing the CNNs empirical cost in Eq 3. We used Stochastic Gradient Descent as discussed in the previous section to minimize the cost function. Here the cost calculated over mini-batches of size 32 is used to approximate the cost over the entire training set. The learning rate *α*_*m*_ is set to 10^−3^ ensuring proper convergence and the scheduling rate which depends on the speed of convergence *τ* is set to 0.9. The training process for both the gating systems LGS and GGS is performed using Adam optimizer [54] with the learning rate of 10^−2^, batch size of 32, *β*_1_ = 0.9, *β*_2_ = 0.999 and decay of 10^−5^.

### Results and discussion

In this experiment, we performed an array of analysis ranging from evaluating the proposed HE-CNN ensemble technique in each stage of DMENet pipeline, comparative evaluation of the DMENet methodology with existing computer-aided solutions, analyzing performance of CNNs vs. proposed HE-CNN technique, comparative study of HE-CNN with other existing ensemble techniques and finally analyzing the performance of DMENet vs. tri-class classification (Grade 0, Grade 1 and Grade 2) solutions. All the analysis and comparisons are made using the evaluation metrics as discussed in the above section.

#### Evaluation of Gamma and Delta Ensembles

**Fig 6:**
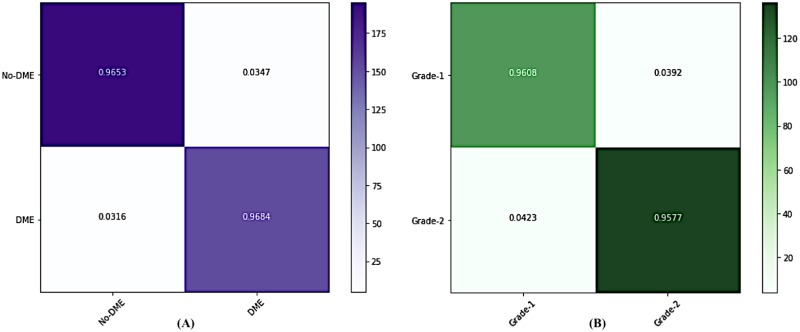
Confusion Matrices showing the performances of (A) Gamma and (B) Delta Ensemble.

**Fig 7:**
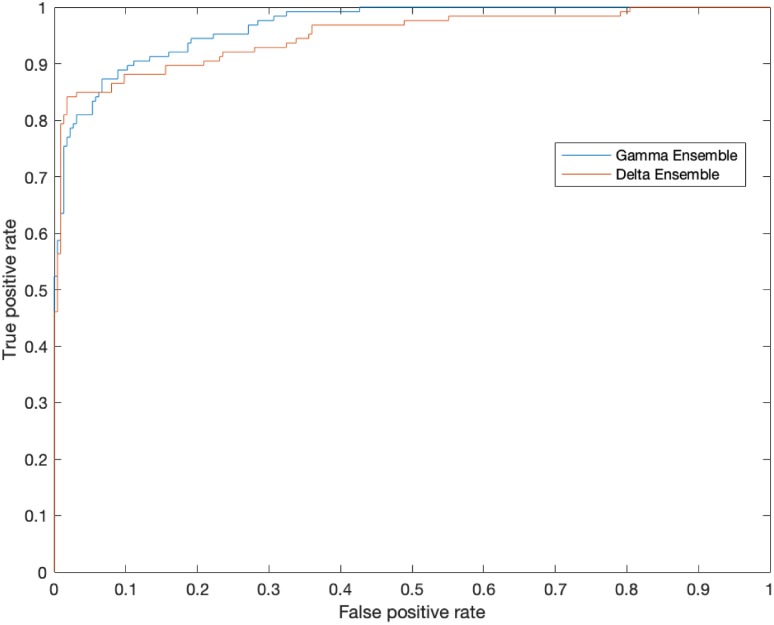
The Receiver Operating Characteristic (ROC) curves demonstrating the performance of Gamma and Delta Ensemble. The area under the curve is 0.9654 in the Gamma Ensemble and 0.9489 in the Delta ensemble.

We demonstrate the performance in each stage of DMENet pipeline using Confusion Matrix and ROC curves. The Confusion Matrix in Fig 6A shows the performance of Gamma Ensemble on 360 test images and Fig 6B gives the performance of Delta Ensemble on 244 test images. Table 3 shows the results obtained by Gamma and Delta ensembles on various metrics of accuracy, specificity,sensitivity, precision, F1−score and Kappa score. The ROC curves of the Gamma and Delta Ensembles are shown in Fig 7. The AUC scores obtained by the Gamma and Delta Ensembles are 0.9654 and 0.9489 respectively. One can clearly see that the Gamma and Delta ensembles achieved promising results.

**Table 3:** Performance of Gamma and Delta Ensembles.

	Gamma Ensemble	Delta Ensemble
Accuracy	96.67	95.91
Specificity	95.63	97.14
Sensitivity	97.50	94.23
Precision	96.53	96.08
F1−score	0.9701	0.9515
Kappa-Score	0.932	0.916

#### Comparative evaluation of HE-CNN methodology

To obtain a benchmark for comparing the performance of HE-CNN ensemble and to show its capability, the proposed model is evaluated with regard to non-ensemble methods using independent CNNs as well as some of the existing ensemble techniques on both the classification tasks. The performance of CNNs using pre-trained ResNet, DenseNet architectures and the results obtained are shown in Table 4. However, one can see from the results that these models do not perform well as they were overfitting the given data. Measures were taken to control overfitting by adding dropout factor of 50% on the fully connected layer before the output in the ResNet as well as the DenseNet archtectures. Though addition of dropout helped in reducing the effect of overfitting, the results obtained are not satisfactory as compared to results given by the proposed HE-CNN ensemble.

**Table 4:** Comparitive study of HE-CNN ensemble.

	Performance Measures			
Classifier	Accuracy	Specificity	Sensitivity	F1−score
***CNN Models***				
ResNet-34	82.16	94.27	70.97	0.7923
ResNet-34+Dropout	83.65	91.38	74.49	0.8216
DenseNet-169	74.21	92.26	58.35	0.7175
DenseNet-169+Dropout	78.43	87.34	64.83	0.7345
***Ensemble Models***				
Ave-Ensemble	83.62	86.42	78.27	0.8132
Soft-Ensemble	84.56	82.84	87.39	0.8421
Pruned-Ensemble	88.78	91.23	88.14	0.8967
Mixture-Ensemble	93.16	92.39	93.94	0.9274
HE-CNN	96.12	95.84	96.32	0.9609

To elaborate the comparative performance of HE-CNN, some standard ensemble techniques have been used as given. Ave-Ensemble averages the output maps of independently trained CNNs to generate the final output [55, 56]. Soft-Ensemble employs several parallel CNN branches, where feature maps are concatenated at the end of each convolutional branch and fully-connected layer processes all the features [57], [58]. Mixture-Ensemble uses multiple CNNs and a weight regulation network [59]. Pruned-Ensemble uses pruning to retain best pre-trained models and then make predictions using maximum voting [60]. The results of these ensemble techniques can be observed in Table 4. All the above models are evaluated independently on two different classification tasks as in DMENet methodology (i.e. DME and severity classification), the results obtained are then mapped to a tri-class problem in order to measure various metrics. This is done in order to understand the true power of HE-CNN architecture. The proposed DMENet approach is compared with other existing methodologies on both the MESSIDOR and IDRiD datasets in Tables 5 and 6 respectively.

**Table 5:** Comparitive study of DMENet’s performance with recent solutions for DME screening using MESSIDOR dataset.

Author	Year	Technique	Results
Lim *et al*. [15]	2011	Marker-controlled watershed transformation for extracting exudates and performed DME stage classification using the location of extracted exudates	Sensitivity-80.9% Specificity-90.2% Accuracy-85.2%
Jaafar *et al*. [16]	2011	Based on top-down image segmentation and local thresholding by a combination of edge detection and region growing Grading of hard exudates was performed using a polar coordinate system centred at the fovea	Sensitivity-93.2% Specificity-90.5%
Akram *et al*. [17]	2012	Filter Bank and SVM classifier	Sensitivity-92.6% Specificity-97.8% Accuracy-97.3%
Baidaa *et al*. [19]	2016	Convolutional Neural Networks	Sensitivity-74.7% Specificity-96.5% Accuracy-88.8%
Li *et al*. [62]	2019	Cross-disease attention network	Accuracy-91.2% Sensitivity-70.8% (classification of both DME and DR)
Proposed DMENet	2019	Hierarchical Ensemble of CNNs (HE-CNN)	Sensitivity-94.68% Specificity-97.19% Accuracy-95.47%

**Table 6:** Comparitive study of DMENet’s performance with recent solutions for DME screening using IDRiD dataset.

Author	Year	Technique	Results
He *et al*. [61]	2019	Auxiliary learning approach and XGBoost classifier	Sensitivity-95.53% Specificity-93.84% Accuracy-94.17%
Li *et al*. [62]	2019	Cross-disease attention network	Joint Accuracy-65.1% (classification of both DME and DR)
Proposed DMENet	2019	Hierarchical Ensemble of CNNs (HE-CNN)	Sensitivity-97.88% Specificity-94.49% Accuracy-96.76%

In the HE-CNN architecture, one of the key components is the gating systems (both LGS and GGS). The adam optimizer is employed for training. We compare SGD with Nesterov momentum (with the learning rate set as 10^−2^ and momentum set as 0.9) to adam optimization method. The results obtained showing superior performance of adam optimization is given below in Table 7.

**Table 7:** Results of optimization methods.

Optimization Method	Accuracy	Specificity	Sensitivity	F1−score
SGD with Nesterov	94.32	93.58	95.92	0.9474
Adam	96.12	95.84	96.32	0.9609

To demonstrate the competence of DMENet pipeline in DME screening, the output of DMENet two-stage pipeline is compared with respect to the tri-class problem. In general, the fundus images used in DME screening are graded into one of the three categories (Grade 0, 1 and 2). The results obtained through the analysis of tri-class classification using pre-trained CNNs as well as ensemble methods using HE-CNN are shown in Table 8 along with results obtained on the DMENet pipeline using the corresponding pre-trained CNN and HE-CNN. The results strongly validates our premise of breaking the tri classification problem into binary classification using two levels. The main advantage of our approach is its consistency over other existing methods on all key metrics, which is extremely important in the medical field. The downside is, that large datasets are not available in the DME testing area and the feasibility of the proposed solution could not be tested in a hospital setting.

**Table 8:** Results of DMENet v/s tri-class classification.

Optimization Method	Accuracy	Specificity	Sensitivity	F1−score
ResNet-34 (Tri-Class)	68.31	83.28	67.21	0.7114
ResNet-34 (DMENet)	82.16	94.27	70.97	0.7923
HE-CNN (Tri-Class)	91.46	88.84	89.62	0.8849
HE-CNN (DMENet)	96.12	95.84	96.32	0.9609

## Conclusions

In this paper, we propose a DMENet methodology for automated screening of DME using raw fundus images. The technique suggested is a two-step pipeline system that achieves effective results. The main contribution of this paper is the usage of an advanced preprocessing technique followed by using the HE-CNN architecture for classification as well as severity grading. Further, a novel loss function is designed to boost the classification performance. The HE-CNN architecture overcomes the problem of overfitting that arises due to the small datasets. The HE-CNN architecture is fairly general and has the ability to address all related classification problems in the field of biomedical imaging with little modification. Our approach outperformed the existing state-of-the-art methods on publicly available databases of IDRiD and MESSIDOR. The viability of our proposed DMENet solution and HE-CNN design has been demonstrated successfully. Our results show that DMENet is resilient to various noisy images and conditions for image acquisition, making it a viable option in low resource settings for the DME screening process. The future plan is to train and validate the proposed methodology by collecting data sets from different medical institutions. The model’s interpretability and explainability is crucial for medical practitioners and other stakeholders to trust the decisions of the proposed technique.

## Supporting information

S1 Dataset (PDF)
