# Supplementary material for: DMENet: Diabetic Macular Edema diagnosis using Hierarchical Ensemble of CNNs
Source: PLoS One. 2020 Feb 10;15(2):e0220677. doi: 10.1371/journal.pone.0220677 (PMC7010263; doi:10.1371/journal.pone.0220677)
Supplement: S1 Dataset — (PDF) [file pone.0220677.s001.pdf]

To,

Anna Mentsl,

We would like to thank you for your queries regarding the datasets used in our research. The response to your queries is given below highlighted in blue colour.

1) *Please confirm the minimal data set underling your study is available at the links you have provided: (http://www.adcis.net/es/descargas-de-software-de-terceros/messidor-es/) and (https://ieee-dataport.org/open- access/indian-diabetic-retinopathy-image-dataset-idrid). Please note, PLOS defines the "minimal data set" to consist of the data set used to reach the conclusions drawn in the manuscript with related metadata and methods, and any additional data required to replicate the reported study findings in their entirety.*

   We have used the datasets in the links given above to reach the conclusion based on our experimentation. We did not use any other data apart from the datasets available in the above links given. Both of them are publicly available datasets.

2) *We note the data available at the link (https://ieee-dataport.org/open-access/indian-diabetic-retinopathy-image- dataset-idrid) requires a login to access the data. Please provide a direct link or DOI to the data which does not require a login to access the data.*

   The IEEE dataport site requires user authentication to download the datasets as part of their security protocol. For your convenience we have provided credentials to access data
   Username: rkscse80@gmail.com
   Password: snu123456

3) *We note the data available at (http://www.adcis.net/es/descargas-de-software-de-terceros/messidor-es/) is through a consortium. In addition, we note under the "Using the Database" section it states, "any unauthorized commercial use are prohibited." Can you please clarify if there is an ethical or legal reasons for this restriction? Please also explain is this was a restriction the authors had to agree to in order to use the data?*

   The Messidor database can be used free of charge, only for research and educational purposes and we have used the database as per the guidelines only for our research purpose. As stated, we have acknowledged the Messidor contributors in our manuscript by citing the article which they have referred to. In order to obtain the dataset we had to fill the form available in the link given, where we had to give the First Name, Last Name, E-mail address, Company/Institution Name, Country and we have agreed to use our information taken in the form as part of Messidor databases download and the support relationship that may result. This is the form http://www.adcis.net/es/descargas-de-software-de-terceros/messidor-es/ which we have submitted.

Kind regards,

Rajeev Kumar Singh
Rohan Gorantla.
